# Neutrophil to lymphocyte ratio in pediatric patients with asthmatic exacerbation and community-acquired pneumonia

**DOI:** 10.1186/s12887-023-04456-6

**Published:** 2023-12-18

**Authors:** Mei Xu, Lingfang Zhou, Jie Zhang, Sha Luo, Yunfeng Zhao, Wei Xiong

**Affiliations:** 1 Department of Pediatrics, Beiwaitan Community Health Service Center, Hongkou District, Shanghai, China; 2 Department of General Practice, Beiwaitan Community Health Service Center, Hongkou District, Shanghai, China; 3 Department of Pediatrics, Central Hospital, Putuo District, Shanghai, China; 4 Department of Pediatrics, Huashan North Hospital, Fudan University, Shanghai, China; 5 Department of Children Healthcare, Maternal and Child Health Hospital, Xuhui District, Shanghai, China; 6 Department of Pulmonary and Critical Care Medicine, Punan Hospital, Pudong New District, Shanghai, China; 7 Department of Pulmonary and Critical Care Medicine, Xinhua Hospital, Shanghai Jiaotong University School of Medicine, No. 1665, Kongjiang Road, Yangpu District, Shanghai, 200092 China

**Keywords:** Neutrophil to lymphocyte ratio, Asthma, Asthmatic exacerbation, Community-acquired pneumonia, Pediatric

## Abstract

**Background:**

Compared with a lower neutrophil to lymphocyte ratio(NLR), a higher one denotes severe asthma exacerbation in hospitalized asthmatic children. In addition, NLR is significantly higher in pediatric patients with community-acquired pneumonia (CAP) than those without. Nevertheless, its role in pediatric patients with concomitant asthmatic exacerbation and CAP remains unknown.

**Methods:**

In this retrospective study including 1032 pediatric patients aged 5 to 14 years old, the diagnostic and prognostic value of NLR in children with concomitant asthmatic exacerbation and non-severe CAP were investigated.

**Results:**

The sensitivity and specificity of NLR for a diagnosis of CAP in patients with asthmatic exacerbation were 56.9% and 90.1%, respectively. The cutoff value of NLR for a diagnosis of CAP in patients with asthmatic exacerbation was 4.15 (P < 0.001). The cumulative asthmatic exacerbation during 3-month followup of patients with high NLR were 23 (21.3%) and 58 (42.0%) in the asthma and asthmatic CAP groups, respectively (P < 0.001). The patients with high NLR who had unimproved CAP were 15 (8.3%) and 23 (12.2%) in the CAP and asthmatic CAP groups, respectively (P = 0.006). Multivariate analyses showed that along with the increase of NLR by 1.0 point, the HR for the occurrence of asthmatic exacerbation and unimproved CAP were 2.91 [1.83–3.96] (P = 0.001) and 3.38 [1.66–5.10] (P < 0.001), respectively.

**Conclusions:**

NLR had high and moderate diagnostic value for the exclusion and indication of CAP, respectively, in pediatric patients with asthmatic exacerbation. It also had prognostic value for the outcomes of pediatric patients with concomitant asthmatic exacerbation and CAP.

## Introduction

Asthma is the most common chronic disease and leading cause of morbidity in childhood, when measured by school absences, emergency department visits, and hospitalizations [1–3]. In addition, community-acquired pneumonia (CAP) remains a common and potentially serious infection in children [4]. It is a leading cause of hospitalization among children in the United States [5].

Neutrophil to lymphocyte ratio (NLR) is a variable with respect to inflammatory status in routine blood test, with a normal range between 0.78 and 3.53 in adult, non-geriatric, and healthy population [6]. It has been validated to be associated with a variety of morbidities mainly including infection [7], solid tumor [8], cardiovascular diseases [9], and pulmonary diseases which mainly comprise chronic obstructive pulmonary disease [10, 11], acute pulmonary embolism [12], acute respiratory distress syndrome (ARDS) [13], lung cancer [14], community-acquired pneumonia [15], and asthma [16].

For children with asthma, NLR is higher in hospitalized asthmatic children with more severe asthma exacerbation compared to non-hospitalized patients or healthy controls [16, 17]. In addition, for children with CAP, NLR is significantly higher in CAP patients than those in the control group [18]. The combined use of NLR and C-reactive protein/mean platelet volume ratios might be adopted in the differential diagnosis of bacterial from viral pneumonia, as well as the prediction of complications in children [19]. Nevertheless, the role of NLR in pediatric asthma patients complicated with CAP has been undefined to date. Accordingly, the current study was performed to explore this subject.

## Methods

### Study design

A retrospective study was performed to determine the role of NLR in children aged 5 to 14 years old with asthmatic exacerbation and non-severe CAP. Pediatric patients with asthmatic exacerbation, those with isolated CAP, and those with concomitant asthmatic exacerbation and CAP who were treated in clinics, emergency departments, and general wards were investigated. Patients of asthma group, CAP group, and asthmatic CAP group were predetermined to be in the approximate proportion of 1:1:1. Asthma group was defined as pediatric asthmatic patients with acute onset without CAP. The diagnoses of asthma and asthmatic exacerbation was established as per the criteria in Global Initiative for Asthma (GINA) [20]. The severity of asthma exacerbation was defined as the mild, moderate, or severe ones without the life-threatening based on the criteria in GINA [20] in the current study. CAP group was defined as pediatric CAP patients without medical history of asthma. The diagnoses of CAP was established as per the guidelines [21]. Asthmatic CAP group was defined as pediatric patients with asthmatic exacerbation and CAP. NLR in the routine blood test assayed at the admission were compared among the aforementioned three groups. The efficiency of baseline NLR for the diagnoses of CAP in patients with asthmatic exacerbation was analyzed. According to the acquired cutoff value of NLR which was 4.15 by using the receiver operator characteristic (ROC) curve analyses, all patients being studied were classified into high and low NLR groups. All patients received corresponding treatment as per the criteria of the guidelines [20, 21].

Then all patients being studied were followed up for three months after their discharge from hospitalization or ambulatory treatment. Routine blood test was assayed again for patients who fulfilled on-site return visit at the 3-month followup visit. Their NLR level at 3-month followup visit and its absolute change and change rate from baseline through endpoint were compared among three groups. The main approach of follow-up was on-site visit, supplemented by telephone visit which was performed for those who could not manage to be on site. Loss to followup was defined as those who neither returned for a followup visit nor were reached by telephone. The cumulative rates of asthmatic exacerbation events in each month were documented for all patients with asthma. For all patients with asthma, the time-to-event rates of asthmatic exacerbation during 3-month follow-up were compared between the asthma and asthmatic CAP groups, as well as between patients with high and low NLR. At the 3-month followup visit, all patients with CAP who had persistent symptoms or signs of CAP underwent a second chest imaging investigation. The rate of unimproved CAP were compared between the CAP and asthmatic CAP groups, as well as between patients with high and low NLR. The correlation between baseline NLR and asthmatic exacerbation during 3-month followup as well as CAP recovery at 3-month followup visit were analyzed for patients in the asthmatic CAP group.

The current study was performed by the investigators of Beiwaitan (North Bund) Community Health Service Center, Putuo District Central Hospital, Huashan North Hospital, Xuhui District Maternal and Child Health Care Hospital, Punan Hospital, and Xinhua Hospital in Shanghai. All data needed for the study were retrieved from the electronic medical records system (EMRS) of each participating hospital. All authors vouch for the completeness and accuracy of the data. No one who was not an author contributed to the writing of manuscript. The study protocol was approved by the institutional review board of each participating hospital.

### Study population

Eligible patients were incorporated into the current study as per the inclusion and exclusion criteria. The inclusion criteria comprised: (1) all eligible patients were 5 to 14 years old; (2) all eligible patients underwent chest computerized tomography (CT) or chest X-ray due to cough, expectoration, chest pain, chest tightness, fever, or dyspnea, to confirm the presence or absence of CAP. The exclusion criteria comprised: (1) patients with severe CAP or respiratory failure who needed to be admitted to the intensive care unit (ICU); (2) patients with differential diagnoses of asthma including chronic upper airway cough syndrome, inhaled foreign body, bronchiectasis, primary ciliary dyskinesia, congenital heart disease, broncopulmonary dysplasia, and cystic fibrosis [20]; (3) patients with differential diagnoses of CAP including acute bronchitis, acute lung injury, congestive heart failure, pulmonary infarction, pneumothorax, acute exacerbation of bronchiectasis, tuberculosis, histoplasmosis, autoimmune disease with lung involvement, pleural empyema, and pulmonary toxicities due to medications [22]; (4) patients with infection other than CAP, solid tumor, cardiovascular diseases, acute pulmonary embolism, and ARDS.

As per the inclusion criteria, 1401 pediatric patients of the participating hospitals from Jan 2012 through Jun 2022 were incorporated into the current study. As per the exclusion criteria, 369 patients were excluded. Finally, a total of 1032 children entered into the analyses. The median age was 9.3 years old. The number of male and female were 636 and 396, respectively.

### Statistical analyses

Measurement data were presented as mean ± standard deviation or median with interquartile range as per if they conformed to normal distribution. Categorical data were presented as percentages. The comparison of measurement data among asthma, CAP, and asthmatic CAP groups were performed by using T-test or analysis of variance (ANOVA). Chi-square test was used to compare the rates. The diagnostic efficiency of NLR for the CAP occurrence in patients with asthma was performed by using ROC curve analyses. The correlation between NLR and the diagnoses of CAP in patients with asthmatic exacerbation was performed by using Logistic regression analyses. Kaplan-Meier method was used to compare the time-to-event rates of cumulative asthmatic exacerbation during 3-month follow-up between patients with high and low NLR for the asthma and asthmatic CAP groups. The correlation between baseline NLR and asthmatic exacerbation during 3-month followup as well as CAP recovery at 3-month followup visit were analyzed by using Cox regression analysis. Statistical analyses was performed by using SPSS 23. Statistical significance was defined as a P value being less than 0.05.

## Results

### Demographics and characteristics of patients

**Fig. 1:**
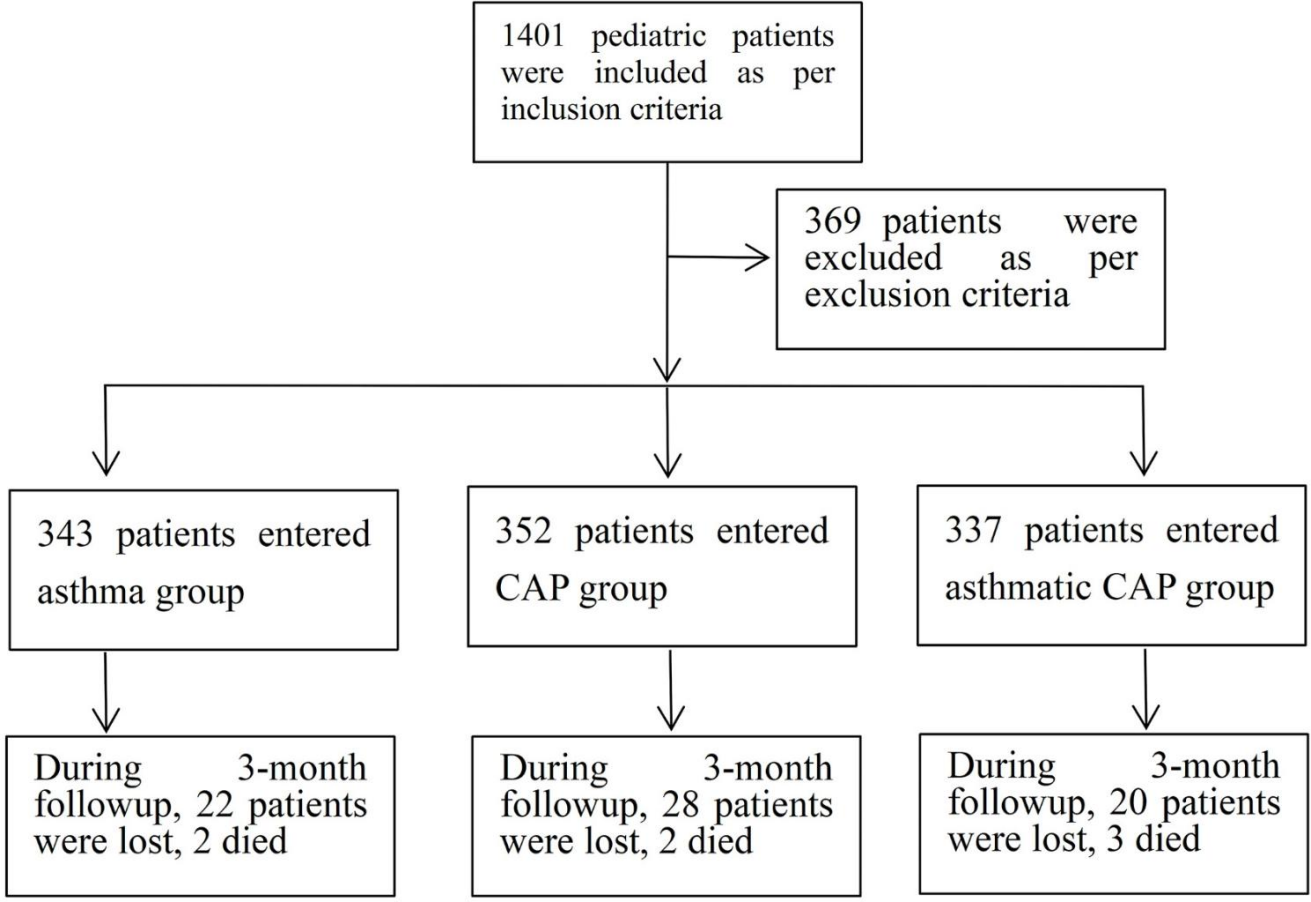
Inclusion, exclusion and follow-up of patients. *Note*: CAP: community-acquired pneumonia

There were 343 patients in the asthma group, 352 patients in the CAP group, and 337 patients in the asthmatic CAP group, respectively. After a 3-month followup, 22, 28, and 20 patients in the asthma, CAP, and asthmatic CAP groups were lost to followup, respectively. No patient died during the treatment in hospitalizations, emergency departments, and clinics. Two and three patients in the asthma and asthmatic CAP groups died from severe exacerbation of asthma during the 3-month followup, respectively. Two patients in the CAP group died from severe recurrent CAP during the 3-month followup. The demographics and clinical characteristics of patients at baseline are summarized in Table 1. The inclusion, exclusion and follow-up of patients are demonstrated in Fig. 1.

**Table 1:** Demographics and characteristics of patients at baseline

Variables	Asthma (n = 343)	CAP (n = 352)	Asthmatic CAP (n = 337)	P value
Age-year	8.1 (6.5–9.7)	9.5 (6.8–12.2)	10.4 (7.3–13.5)	0.532
Gender (male sex)-no. (%)	195 (56.9)	232 (65.9)	209 (62.0)	0.683
Age of asthma onset-no.	14.6 (10.2–19.0)		16.2 (11.8–20.6)	0.884
Times of oral glucocorticoid in the previous year-no.	2.5 (1.7–3.3)		2.8 (1.9–3.7)	0.762
Emergency department visits in the previous year-no.	3.6 (2.1–5.1)	1.4 (1.0−1.8)	3.1 (2.2−4.0)	0.001
Hospitalizations in the previous year-no.	0.5 (0.3–0.7)	0.2 (0.0−0.4)	0.7 (0.4−1.0)	0.001
Percentage of asthma-control days-%	86.4 (78.3–94.5)		83.2 (72.8–93.6)	0.831
Albuterol inhalations per week-no.	1.6 (0.5–2.7)		1.9 (1.0−2.8)	0.673
Use of ICS in the previous year- no. (%)	230 (67.1)		243 (72.1)	0.516
Use of LTRA in the previous year-no. (%)	93 (27.1)		66 (19.6)	0.005
Blood eosinophil count-cells/mm^3^	266.3 (176.5−356.1)	129.4 (49.8–209.0)	237.6 (127.2–348.0)	0.001
IgE-kU/L	72.0 (36.9−107.1)	41.2 (22.8–59.6)	66.8 (41.6–92.0)	0.001
NLR	2.3 (1.0−3.6)	2.5 (1.2–3.8)	3.5 (2.0–5.0)	0.001
Oxygen saturation-%	96.7 (93.5–99.9)	97.2 (95.1–99.3)	95.4 (92.8–98.0)	0.892
PaO_2_-mmHg	75.3 (62.9–87.7)	73.6 (64.5–82.7)	71.8 (58.4–85.2)	0.573
**Clinical setting**				
Clinics	57 (16.6)	56 (15.9)	45 (13.3)	0.125
Emergency department	105 (30.6)	101 (28.7)	96 (28.5)	
General wards	181 (52.8)	195 (55.4)	196 (58.2)	

*Note*: CAP: community-acquired pneumonia, ICS: inhaled glucocorticoids, LTRA: leukotriene receptor antagonist, NLR: neutrophil to lymphocyte ratio, PaO_2_: partial pressure of oxygen

### Comparison of NLR among asthma, CAP, and asthmatic CAP groups at baseline

The median NLR of the asthma, CAP, and asthmatic CAP groups at the admission of patients with each disease were 2.3 (95% confidence interval [CI][1.0-3.6]), 2.5 (95%CI[1.2–3.8]), 3.5 (95%CI[2.0–5.0]), respectively. The NLR of the patients in the asthmatic CAP group was significantly higher than those of the patients in the asthma and CAP groups (P = 0.001). No significant difference was found with respect to NLR between the asthma and CAP groups (P = 0.874) (Table 1).

### Comparison of NLR and NLR change at 3-month among asthma, CAP, and asthmatic CAP groups

At the 3-month followup visit, 112, 95, and 124 patients in the asthma, CAP, and asthmatic CAP groups underwent another routine blood test. Their NLR in the asthma, CAP and asthmatic CAP groups were 1.8 (0.9–2.7), 1.2 (0.5–1.9) and 2.2 (1.3–3.1), respectively (P = 0.009). Their NLR change from baseline through endpoint in the asthma, CAP and asthmatic CAP groups were 1.1 (0.4–1.8), 1.8 (0.9–2.7), and 0.6 (0.2-1.0), respectively (P < 0.001). The change rate of NLR from baseline through endpoint in the asthma, CAP and asthmatic CAP groups were 37.9%, 60.0%, and 21.4%, respectively (P < 0.001).

### Diagnostic efficiency of NLR for the diagnoses of CAP in patients with asthma

**Fig. 2:**
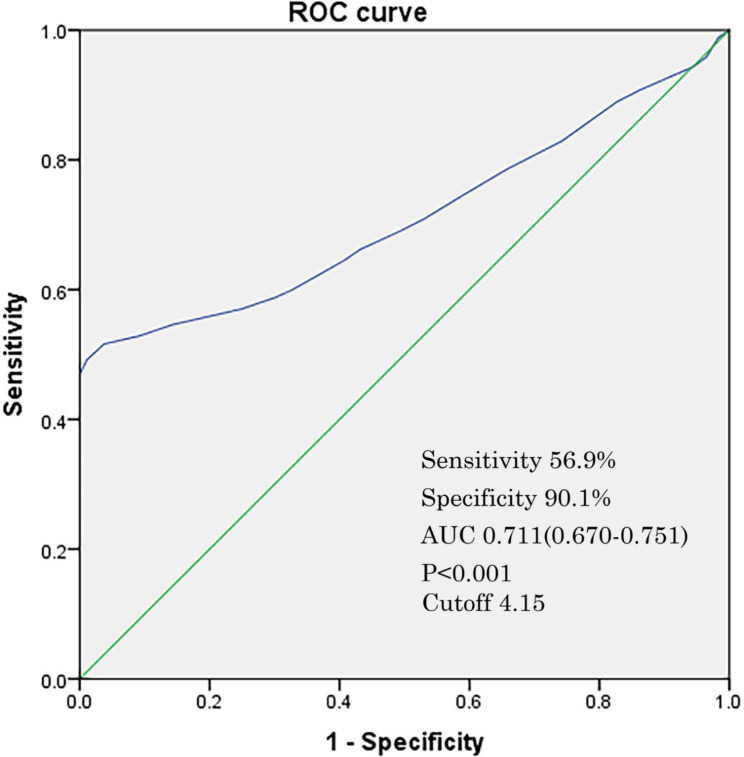
ROC curve analysis of efficiency of NLR for diagnoses of CAP in patients with asthma. *Note*: ROC: receiver operator characteristic, NLR: neutrophil to lymphocyte ratio, CAP: community-acquired pneumonia; AUC: area under curve

After a ROC curve analysis, the results showed that the sensitivity and specificity of NLR for a diagnosis of CAP in pediatric patients with asthma were 56.9% and 90.1%, respectively. The positive predictive values (PPV) and negative predictive values (NPV) were 85.1% and 67.7%, respectively. The Youden index was 0.470. The area under curve (AUC) of NLR for a diagnosis of CAP in pediatric patients with asthma was 0.711 (0.670–0.751). The cutoff value of NLR for a diagnosis of CAP in pediatric patients with asthma was 4.15 (P < 0.001) (Fig. 2). A Logistic regression analysis demonstrated that, for pediatric patients with asthmatic exacerbation, the one with NLR higher than the cutoff value had higher risk to develop a CAP, than those with NLR lower than the cutoff value (OR 2.5 [1.4–3.6], P < 0.001).

### Cumulative asthmatic exacerbation during 3-month followup

**Fig. 3:**
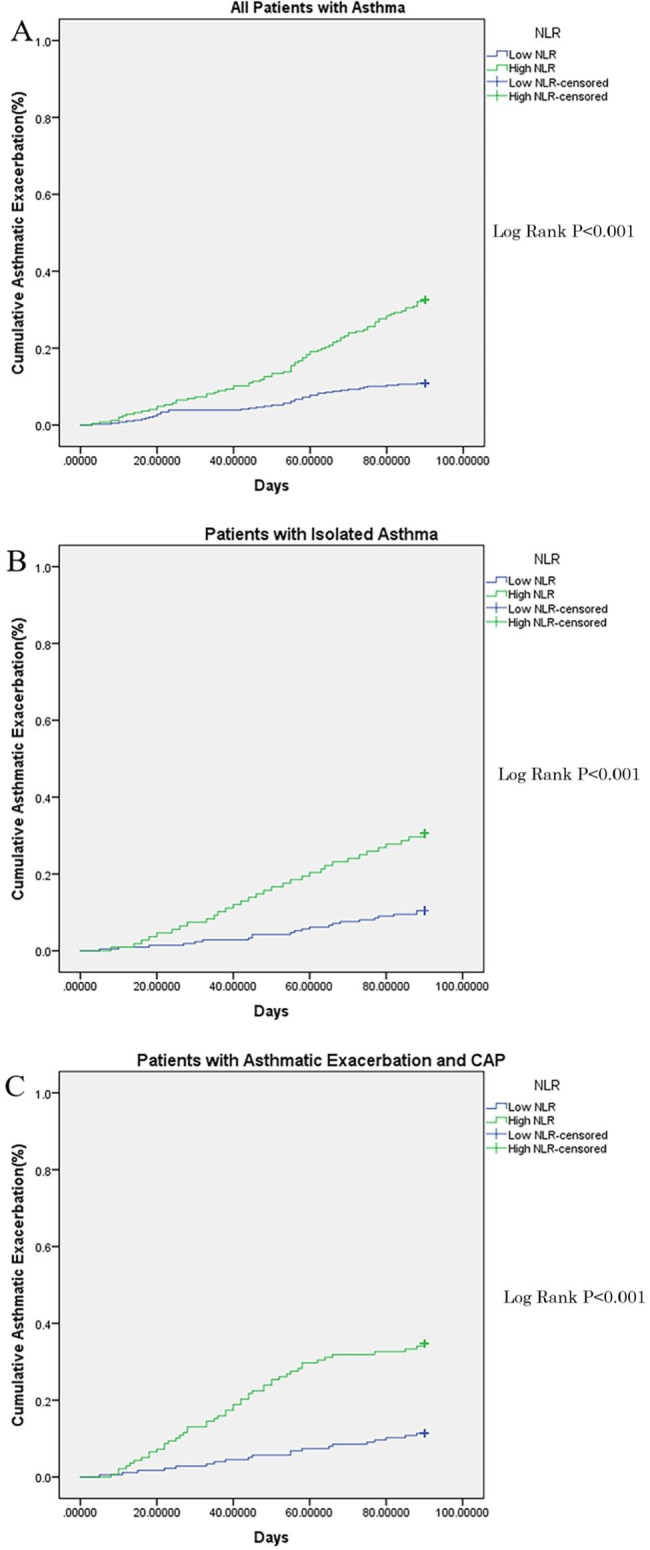
Cumulative asthmatic exacerbation between high and low NLR patients with asthma during the followup. *Note*: NLR: neutrophil to lymphocyte ratio, CAP: community-acquired pneumonia

After the deduction of patients who were lost to followup and who died during followup, 319 and 314 patients in the asthma and asthmatic CAP groups finished the entire followup. The cumulative asthmatic exacerbation events during 3-month followup in the asthma and asthmatic CAP groups were 45 (14.1%) and 78 (24.8%), respectively (P < 0.001). According to the acquired cutoff value of baseline NLR which was 4.15, all the patients with asthma being followed up were classified into the high (n = 246) and low (n = 387) NLR groups. The number of patients with high and low NLR were 108 and 211 in the asthma group, respectively, whereas those with high and low NLR were 138 and 176 in the asthmatic CAP group, respectively (P < 0.001). For all patients who had asthma, the cumulative asthmatic exacerbation during 3-month followup in the high and low NLR groups were 81 (32.9%) and 42 (10.9%), respectively (P < 0.001) (Panel A, Fig. 3). For patients in the asthma group, the cumulative asthmatic exacerbation during 3-month followup in the high (n = 108) and low (n = 211) NLR groups were 23 (21.3%) and 22 (10.4%), respectively (P < 0.001) (Panel B, Fig. 3). For patients in the asthmatic CAP group, the cumulative asthmatic exacerbation events during 3-month followup in the high (n = 138) and low (n = 176) NLR groups were 58 (42.0%) and 20 (11.4%), respectively (P < 0.001) (Panel C, Fig. 3). The cumulative asthmatic exacerbation events during 3-month followup of patients with high NLR were 23 (21.3%) and 58 (42.0%) in the asthma and asthmatic CAP groups, respectively (P < 0.001). The cumulative asthmatic exacerbation events during 3-month followup of patients with low NLR were 22 (10.4%) and 20 (11.4%) in the asthma and asthmatic CAP groups, respectively (P = 0.367).

### CAP recovery for all patients with CAP at 3-month

After the deduction of patients who were lost to followup and who died during followup, 322 and 314 patients in the CAP and asthmatic CAP groups fulfilled the entire followup. The number of patients with persistent symptoms and signs of CAP were 32 (9.9%) and 60 (19.1%) in the CAP and asthmatic CAP groups, respectively (P < 0.001). After a second chest imaging investigation, 21 (6.5%) and 36 (11.5%) patients presented with unimproved CAP on chest imaging in the CAP and asthmatic CAP groups, respectively (P = 0.029).

**Fig. 4:**
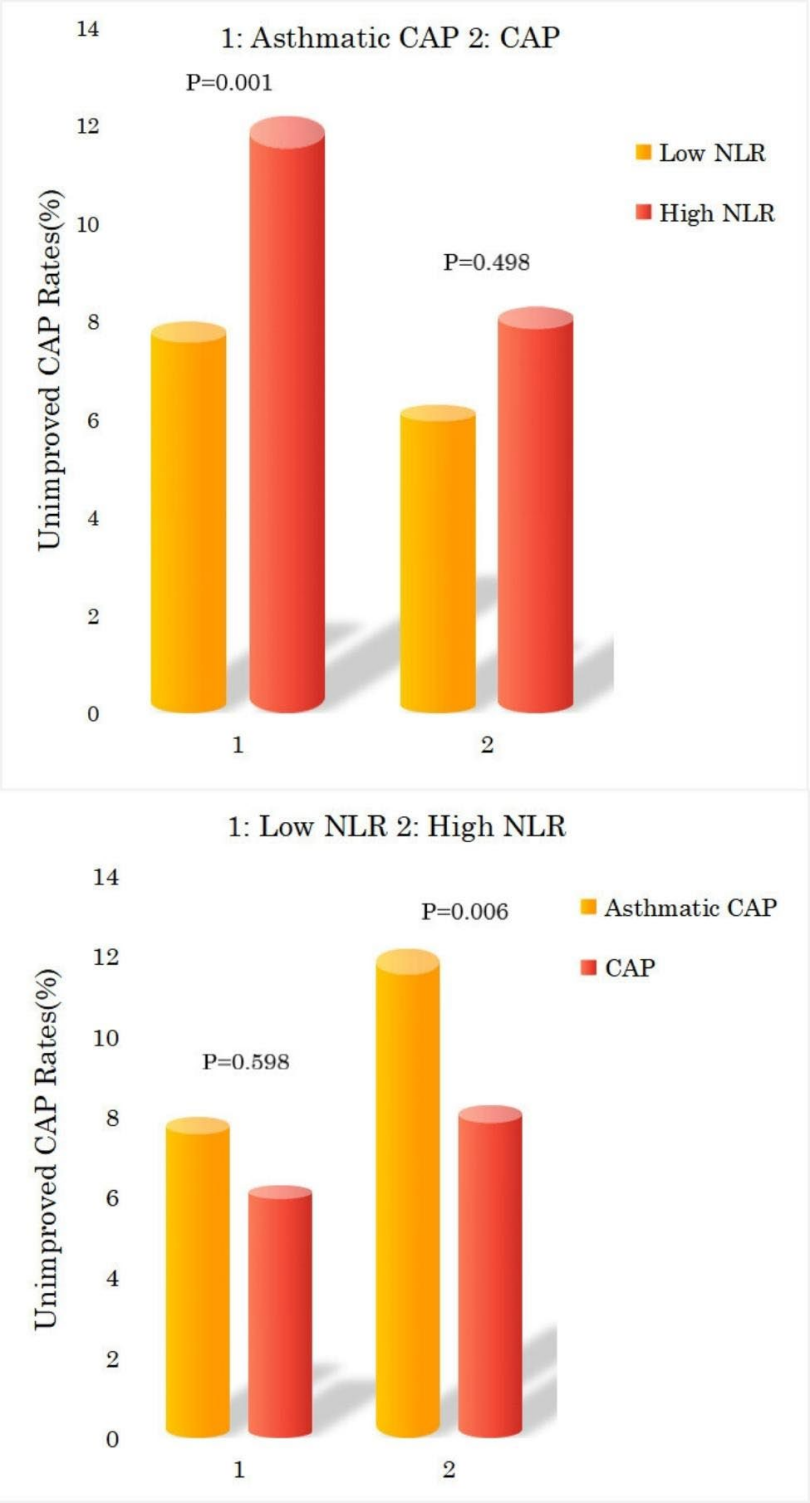
Unimproved CAP rates between high and low NLR, CAP and asthmatic CAP groups. *Note*: CAP: community-acquired pneumonia; NLR: community-acquired pneumonia; High NLR: ≥4.15; Low NLR: <4.15

According to the acquired cutoff value of baseline NLR which was 4.15, all the patients who had CAP were classified into the high (n = 369) and low (n = 267) NLR groups. The number of patients with high and low NLR were 180 and 142 in the CAP group, respectively, whereas those with high and low NLR were 189 and 125 in the asthmatic CAP group, respectively (P = 0.273). For all patients who had CAP, the number of patients presented with unimproved CAP on chest imaging were 38 (10.3%) and 19 (7.1%) in the high and low NLR groups, respectively (P = 0.001). For the CAP group, the patients who had unimproved CAP on chest imaging were 15 (8.3%) and 9 (6.3%) in the high (n = 180) and low (n = 142) NLR groups, respectively (P = 0.498). For the asthmatic CAP group, the patients who had unimproved CAP on chest imaging were 23 (12.2%) and 10 (8.0%) in the high (n = 189) and low (n = 125) NLR groups, respectively (P = 0.001). The patients with high NLR who had unimproved CAP on chest imaging were 15 (8.3%) and 23 (12.2%) in the CAP and asthmatic CAP groups, respectively (P = 0.006). The patients with low NLR who had unimproved CAP on chest imaging were 9 (6.3%) and 10 (8.0%) in the CAP and asthmatic CAP groups, respectively (P = 0.598). The unimproved CAP rates between high and low NLR groups, CAP and asthmatic CAP groups are demonstrated in Fig. 4.

The number of patients with both unimproved CAP and cumulative asthmatic exacerbation in the asthmatic CAP group was 27 (8.6%). Among these 27 patients, 20 (74.1%) ones had high NLR, whereas 7 (25.9%) ones had low NLR (P < 0.001).

### Correlation between baseline NLR and asthmatic exacerbation as well as CAP recovery for the asthmatic CAP group

For the asthmatic CAP group, an univariate analysis with respect to the correlation between baseline NLR and asthmatic exacerbation during 3-month followup demonstrated that, along with the increase of NLR by 1.0 point, the hazard ratio (HR) for the occurrence of asthmatic exacerbation was 2.78 [1.66–3.90] (P = 0.001). After incorporating age and sex, a multivariate analysis showed that along with the increase of NLR by 1.0 point, the HR for the occurrence of an asthmatic exacerbation was 2.91 [1.83–3.96] (P = 0.001).

An univariate analysis with respect to the correlation between baseline NLR and CAP recovery at 3-month demonstrated that, along with the increase of NLR by 1.0 point, the hazard ratio (HR) for the occurrence of unimproved CAP was 3.14 [1.32–4.96] (P < 0.001). After incorporating age and sex, a multivariate analysis showed that along with the increase of NLR by 1.0 point, the HR for the occurrence of unimproved CAP was 3.38 [1.66–5.10] (P < 0.001).

## Discussion

To our best knowledge, this is the first study with respect to such topic, since comparable studies that can be identified are rare to date. Accordingly, it is intractable to compare the current study with previous ones. A related study by Cag et al. investigated the significance of NLR in 991 consecutive patients aged 5 years or older who were diagnosed with asthma, patients with lower respiratory tract (LRT) infections had a greater NLR (2.06 [1.32–3.26]), compared with patients without LRT infections (1.55 [1.09–2.40]) (P < 0.001) [23]. The result of the present study is basically consistent with that of the study of Cag et al. [23] in this regard.

In a previous retrospective study, mean NLR (2.07 ± 1.41) in the study group (n = 469) which was defined as pediatric patients with asthma was higher than that (1.77 ± 1.71) in the control group (n = 170) which was defined as pediatric patients without evidence of allergic disease or infection (p = 0.043). Mean NLR was weakly positively correlated with the number of hospitalizations (p = 0.012). The percentage of eosinophils was negatively correlated with NLR (p = 0.001). NLR could be a biomarker for the assessment of systemic inflammation in asthmatic patients [17]. In another retrospective cross-sectional study, 91 hospitalized children due to more severe asthma exacerbation were compared with 120 non-hospitalized children with moderate to severe asthma exacerbation. An ROC curve analysis revealed that the cutoff value of NLR was 2.52 for the prediction of hospitalization. In a multivariate analysis, NLR ≥ 2.52 (OR 2.13, 95% CI 1.09–4.14; P = 0.027) was associated with hospitalization. NLR was higher in hospitalized children with more severe asthma exacerbation compared to non-hospitalized patients with moderate to severe asthma exacerbation [16]. NLR may be considered as an indicator for severity assessment of asthma exacerbation. In another retrospective study consisting of 114 pediatric patients with CAP and 50 control subjects, NLR level was significantly higher in the CAP group than that in the control group (p < 0.001) [18]. NLR may be regarded as a predictor for the development of pediatric CAP. These finding are basically consistent with those of the present study.

In the current study, we further confirmed that NLR had a high diagnostic value for excluding CAP occurrence and a moderate value for suggesting CAP occurrence in pediatric patients with asthmatic exacerbation. Moreover, for pediatric patients with concomitant asthmatic exacerbation and CAP, cumulative asthmatic exacerbation events in the next three months were more than that in the asthma group. In addition, the cumulative asthmatic exacerbation events of patients with high NLR in the asthmatic CAP group were more than that in the asthma group. Moreover, the patients in the asthmatic CAP group presented with more unimproved CAP, compared with the patients with isolated CAP. The patients with high NLR in the asthmatic CAP group had more unimproved CAP than those in the CAP group. Albeit per the results of some previous studies, the use of inhaled glucocorticoids (ICS) may be accountable for the unsatisfactory CAP improvement in the asthmatic CAP group [24, 25], other studies indicated that ICS use was not associated with an increase of pneumonia [26, 27]. Notwithstanding systemic glucocorticoid which could be used in asthmatic patients may induce leukocytosis [28] thereby affecting NLR, most of the asthmatic patients merely received long-term inhaled steroids therapy in the current study, whereas very few (0.8%) asthmatic patients received long-term systemic steroids therapy. For children who underwent systemic steroids during acute asthmatic attack, NLR was collected at the moment when the children were admitted prior to the use of systemic steroids. As such, NLR was hardly affected by glucocorticoids in the current study.

In short, the high the NLR or the more complicated the diseases, the more the asthmatic exacerbation or unimproved CAP. Patients with high NLR in the asthmatic CAP group had the most asthmatic exacerbation and unimproved CAP. Since NLR is a reflection of the inflammatory status of patients, the combination of asthmatic exacerbation and CAP may lead to a further increase in NLR. The chest imaging review of CAP patients 3 months later indicated that there were still a small number of patients with incomplete absorption of lung lesions, most of which (85%) were organizing pneumonia or fibrous shadow secondary to CAP, and a few of them (15%) were recurrent pneumonia. It suggests that an elevated inflammatory state may result in not only frequent asthma exacerbation but also focal delayed absorption and recurrence of CAP than patients with isolated asthmatic exacerbation or CAP, respectively, for pediatric patients with concomitant asthmatic exacerbation and CAP. Since a high baseline NLR implies a high expression of inflammatory response state in children with either asthma or CAP, it is understandable that this may result in frequent asthmatic exacerbation and/or non-regression of CAP.

The current cutoff of NLR has a high specificity but a moderate sensitivity in the pretest assessment of likelihood of CAP in pediatric patients with asthmatic exacerbation. This means that NLR is better to rule out than to indicate CAP in children with asthmatic exacerbation. It suggests that such diagnostic criteria may yield low misdiagnoses, whereas may lead to moderate missed diagnoses. Of note, the reported incidence of radiographic CAP in pediatric patients with asthmatic exacerbation is less than 5% [29] which is not very considerable. In this context, misdiagnosis rather than missed diagnosis is more likely to occur during the diagnostic examination. The primary purpose of pretest assessment of CAP in children with asthmatic exacerbation should be more focusing on ruling out the possibility of CAP than indicating its possibility, in order to avoid unnecessary radiation. For this reason, such threshold value of NLR can be considered as an eligible indicator for the pretest assessment of CAP occurrence in children with asthmatic exacerbation.

Such findings may derive certain clinical implications. When encountering pediatric patients with asthmatic exacerbation in daily clinical practice, pediatricians should pay attention to patients’ NLR in routine blood test. It is recommended that a CAP could be basically excluded when NLR is less than 4.15, otherwise CAP may be considered when NLR exceeds 4.15. In addition, for asthmatic patients complicated with CAP, it is necessary to follow them up closely for the potential episodes of asthmatic exacerbation and unimproved CAP.

Several limitations have to be acknowledged for the current study. First of all, it is a retrospective study. Prospective studies are warranted in the future. Secondly, since the NLR measurement at the 3-month was performed in part of the whole study population, the small sample may not reflect the true nature of NLR changes in this patient population. Thirdly, since CAP screening was not routinely performed for patients with isolated asthma during the followup, it was intractable to determine whether any of these patients may have a later new CAP. Nevertheless, records showed that only 5 patients with isolated asthma had a history of later CAP during the followup. Fourthly, the results may not be applicable to pediatric patients who are younger than 5 years old, since those patients were not incorporated into the current study. Last but not least, the results may not be applicable to patients with severe CAP or respiratory failure who require admission to ICU, since those patients were not incorporated into the current study either.

**Fig. 5:**
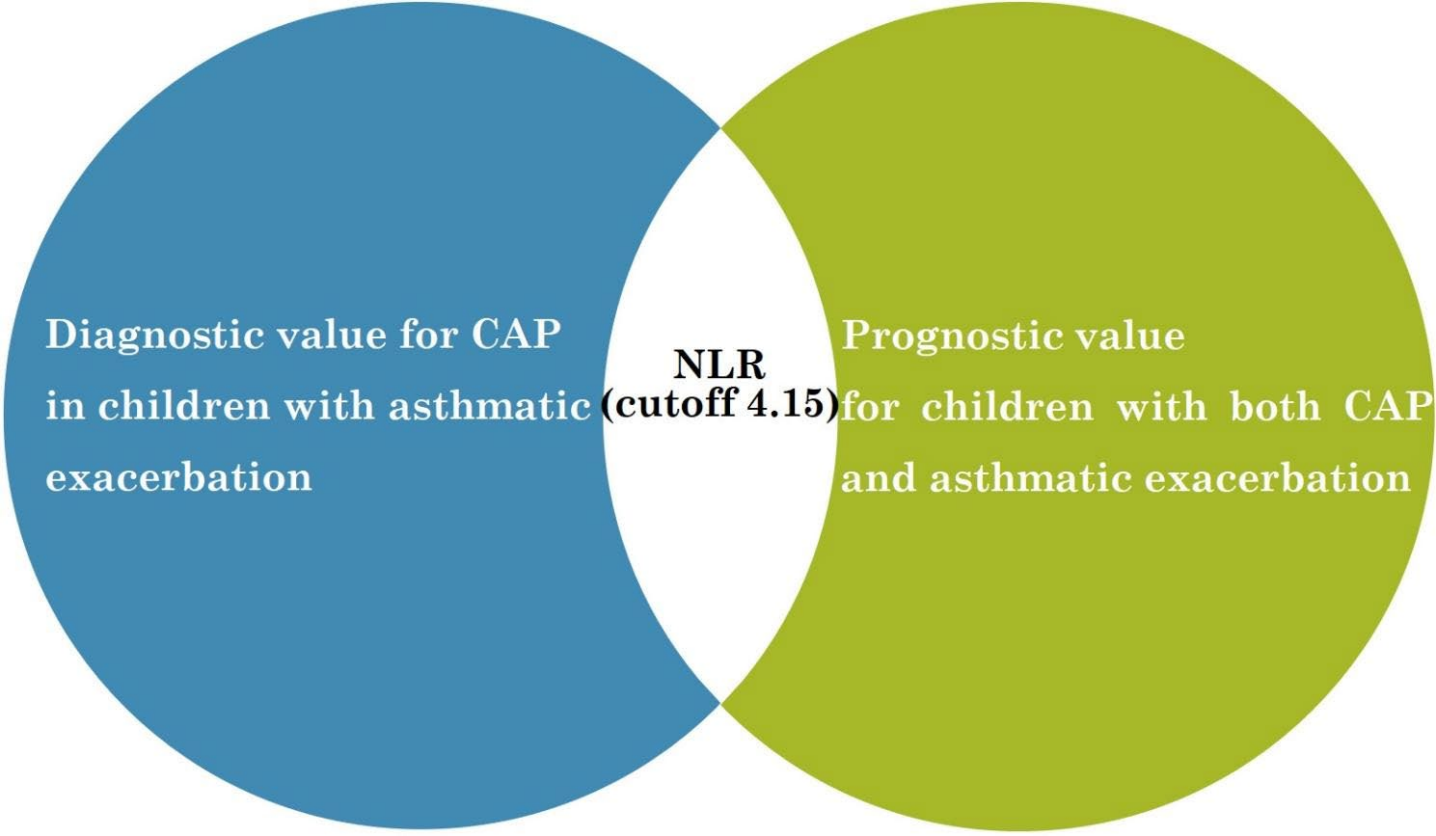
A Venn diagram of a summary of the results. *Note*: NLR: neutrophil to lymphocyte ratio, CAP: community-acquired pneumonia

In conclusion, the current study revealed that pediatric patients with both asthmatic exacerbation and CAP had higher NLR than those with isolated asthmatic exacerbation or CAP. NLR with a cutoff of 4.15 had high diagnostic value for the exclusion of CAP, whereas had moderate diagnostic value for the indication of CAP in pediatric patients with asthmatic exacerbation. Meanwhile, NLR also had prognostic value for the outcomes of pediatric patients with both asthmatic exacerbation and CAP. As illustrated in the Venn Diagram of Fig. 5, NLR is either a diagnostic factor for CAP occurrence in children with asthmatic exacerbation, or a prognostic factor for outcomes of children with both asthmatic exacerbation and CAP. These findings may have certain clinical implications in the concerned patient population.

## Data Availability

The datasets used and/or analyzed during the current study are available from the corresponding author on reasonable request.
